# Variability of the QuantiFERON®-TB Gold In-Tube Test Using Automated and Manual Methods

**DOI:** 10.1371/journal.pone.0086721

**Published:** 2014-01-23

**Authors:** William C. Whitworth, Donald J. Goodwin, Laura Racster, Kevin B. West, Stella O. Chuke, Laura J. Daniels, Brandon H. Campbell, Jamaria Bohanon, Atheer T. Jaffar, Wanzer Drane, Paul A. Sjoberg, Gerald H. Mazurek

**Affiliations:** 1 Division of Tuberculosis Elimination, Centers for Disease Control and Prevention, Atlanta, Georgia, United States of America; 2 Epidemiology Services Branch, United States Air Force School of Aerospace Medicine, Brooks City-Base, Texas, United States of America; 3 Department of Occupational Medicine/TB Prevention/Deployment Medicine, Wilford Hall Medical Center, Reid Clinic, Lackland Air Force Base, Texas, United States of America; 4 Northrop Grumman Information Systems Sector, Atlanta, Georgia, United States of America; 5 CDC Foundation, Atlanta, Georgia, United States of America; 6 Professor Emeritus of Biostatistics, University of South Carolina, Columbia, South Carolina, United States of America; 7 Epidemiology Consult Services, United States Air Force School of Aerospace Medicine, Wright-Patterson Air Force Base, Dayton, Ohio, United States of America; Public Health Agency of Barcelona, Spain

## Abstract

**Background:**

The QuantiFERON®-TB Gold In-Tube test (QFT-GIT) detects *Mycobacterium tuberculosis* (*Mtb*) infection by measuring release of interferon gamma (IFN-γ) when T-cells (in heparinized whole blood) are stimulated with specific *Mtb* antigens. The amount of IFN-γ is determined by enzyme-linked immunosorbent assay (ELISA). Automation of the ELISA method may reduce variability. To assess the impact of ELISA automation, we compared QFT-GIT results and variability when ELISAs were performed manually and with automation.

**Methods:**

Blood was collected into two sets of QFT-GIT tubes and processed at the same time. For each set, IFN-γ was measured in automated and manual ELISAs. Variability in interpretations and IFN-γ measurements was assessed between automated (A1 vs. A2) and manual (M1 vs. M2) ELISAs. Variability in IFN-γ measurements was also assessed on separate groups stratified by the mean of the four ELISAs.

**Results:**

Subjects (N = 146) had two automated and two manual ELISAs completed. Overall, interpretations were discordant for 16 (11%) subjects. Excluding one subject with indeterminate results, 7 (4.8%) subjects had discordant automated interpretations and 10 (6.9%) subjects had discordant manual interpretations (p = 0.17). Quantitative variability was not uniform; within-subject variability was greater with higher IFN-γ measurements and with manual ELISAs. For subjects with mean TB Responses ±0.25 IU/mL of the 0.35 IU/mL cutoff, the within-subject standard deviation for two manual tests was 0.27 (CI_95_ = 0.22–0.37) IU/mL vs. 0.09 (CI_95_ = 0.07–0.12) IU/mL for two automated tests.

**Conclusion:**

QFT-GIT ELISA automation may reduce variability near the test cutoff. Methodological differences should be considered when interpreting and using IFN-γ release assays (IGRAs).

## Introduction

The QuantiFERON®-TB Gold In-Tube test (QFT-GIT) was designed to detect *Mycobacterium tuberculosis* (*Mtb*) infection by quantifying the amount of interferon-γ (IFN-γ) released when whole blood is stimulated with specific *Mtb* antigens [1]. The amount of IFN-γ released is determined by enzyme-linked immunosorbent assay (ELISA). QFT-GIT and other IFN-γ release assays (IGRAs) are alternatives to the tuberculin skin test (TST) for detecting *Mtb* infection, both latent infection (LTBI) and infection manifesting as active disease. However, variability may limit QFT-GIT utility. Serial testing of healthcare workers (HCW) has demonstrated higher than expected QFT-GIT conversion and reversion rates in low-prevalence settings [2]–[17]. In addition, comparisons of simultaneously-performed second- and third-generation QuantiFERON IGRAs have demonstrated greater than expected interpretative discordance [18], [19]. Assessments of QFT-GIT repeatability and reproducibility have demonstrated appreciable amounts of variability [20], [21].

Estimates of variability have varied widely among studies that used different methods of performing QFT-GIT, different indices to assess variability, and different study populations with varied prevalence of *Mtb* infection and risk of infection. QFT-GIT variability in published studies has been attributed to temporal biologic fluctuations within subjects due to new *Mtb* infection [2], [22], progression or treatment of human immunodeficiency virus (HIV) infection [23], response to treatment [24]–[27], differences in testing methods (such as difference in delay to incubation, duration of incubation, or incubation temperature) [14], [28], [29], and nonspecific test fluctuations due to random variation [2]–[4], [21], [30]. Determination of the background variability (noise, a change beyond which represents a “true” change) is challenging, especially near the cutoff separating positive and negative test interpretations. This is of critical importance in detecting new infection.

QFT-GIT is a complex test and may be prone to nonspecific random variation. Technical errors attributable to test complexity appear to contribute to IGRA variability [19]. Few studies have assessed the nonspecific random variability of QFT-GIT when repeated on the same samples or samples collected at the same time using identical methods. Discordance in interpretation when QFT-GIT was repeated on the same sample in different ELISAs has been approximately 3.6% [2], [10] and 8.0% to 8.3% [29], [31] when repeated in the same ELISA.

Although the development and initial evaluation of QFT-GIT relied on manual ELISA methods, automation may reduce QFT-GIT variability. Of the 126 measurements required for one QFT-GIT, 115 are automatable (Goodwin et. al., manuscript in preparation). To our knowledge, a comparison of variability between tests performed manually and between tests performed using an automated workstation has not been reported. To assess the impact of ELISA automation on QFT-GIT, we compared test results and measured variability when tests were performed with manual and automated methodologies.

## Methods

### Ethics Statement

The Centers for Disease Control and Prevention (CDC) and Wilford Hall Medical Center human subjects institutional review boards approved this study. All subjects provided written informed consent.

### Subject Selection

After obtaining approval from human subjects review boards at the Centers for Disease Control and Prevention (CDC, Protocol # 5078) and Wilford Hall Medical Center (U.S. Air Force (USAF), Protocol # FWH20080002H), subjects were recruited from among CDC and USAF staff located in Atlanta, Georgia, and San Antonio, Texas, respectively, as part of a larger study investigating QFT-GIT variability. To increase the proportion of subjects with positive QFT-GIT results and to assess subjects with a continuous range of IFN-γ measurements (including those with IFN-γ measurements near the cutoff separating positive and negative interpretations), only persons with self-reported prior positive TST results were recruited. Prior unpublished assessments among a similar cohort found that 40% to 50% of persons with self-reported prior positive TST results were positive by QFT-GIT. Exclusion criteria were age of less than 18 years or a history of a severe TST reaction (e.g., blistering, scarring, or anaphylaxis). All subjects provided informed written consent and completed a detailed study questionnaire.

### QFT-GIT Procedure

Blood from each subject was collected at one morning visit into two sets of QFT-GIT tubes (Set 1 or Set 2) so that an automated ELISA and a manual ELISA could be performed from each set of tubes. Tubes were purchased from Cellestis, Ltd (Cellestis Limited, Carnegie, Victoria, Australia), and each set of tubes included a Nil tube, a TB antigen tube, and a Mitogen tube. Each tube was labeled with a number and a barcode that (1) identified the specimen, (2) identified the tube type (i.e., Nil tube, TB antigen tube, or Mitogen tube), and (3) linked the specimen to subject and collection information. One mL of blood was collected into each tube and tube contents were mixed with a Stuart rock and roll mixer (SciTech Instruments, Inc., Franklin, NJ) for 3 minutes at 33 RPM. Within one hour of blood collection, tubes were incubated at 37±0.5°C for 23 to 24 hours and then centrifuged at 3,000 g for 10 minutes.

IFN-γ concentrations in plasmas in Nil tubes (Nil), TB antigen tubes (TB), and Mitogen tubes (Mitogen) were determined by ELISAs performed on the day after blood collection using reagents included in QFT-GIT kits. ELISAs were performed with the aid of an automated ELISA workstation (automated ELISA) or without the aid of an automated ELISA workstation (manual ELISA). Triturus automated ELISA workstations (Grifols, USA, Inc., Miami, FL) were used in CDC and USAF labs. For manual ELISAs, reagents were dispensed with Rainin LTS single and multichannel pipetters (Rainin Instrument, LLC, Oakland, CA); plates were washed with a Biotrak II Microplate washer (Biochrom, Ltd., Cambridge, UK) in the CDC lab and a Dynex Ultrawash Plus Microplate washer (Dynex Technologies, Chantilly, VA) in the USAF lab; and optical densities (ODs) were measured with a Thermo Scientific, Multiskan Ascent (Waltham, MA) in the CDC lab and a BioTek ELX800 microplate reader (BioTek Instruments, Inc., Winooshi, VT) in the USAF lab. IFN-γ standards from QFT-GIT kits were serially diluted and eight IFN-γ concentrations (i.e., 8, 4, 2, 1, 0.5, 0.25, 0.125, and 0 IU/mL) were used in duplicate to create a standard curve for each ELISA. OD values were imported electronically, and plasma IFN-γ concentrations were determined using a Microsoft Access database (Microsoft, Inc., Seattle, WA) developed at CDC. ELISAs not meeting quality specifications as defined by the manufacturer [32] were immediately repeated. TB Responses were calculated by subtracting Nil from TB, and Mitogen Responses were calculated by subtracting Nil from Mitogen.

Test results were interpreted as indicated in the CDC guidelines and Cellestis package insert [1], [32]. The interpretation was “positive” if the Nil was ≤8.0 IU/mL and the TB Response was ≥0.35 IU/mL and ≥25% of the Nil. The interpretation was “negative” if the Nil was ≤8.0 IU/mL, the Mitogen Response was ≥0.5 IU/mL, and the TB Response was <0.35 IU/mL or <25% of the Nil. The interpretation was “indeterminate” if (*1*) the Nil was >8.0 IU/mL or (*2*) the Nil was ≤8.0 IU/mL, the Mitogen Response was <0.5 IU/mL, and the TB Response was <0.35 IU/mL or <25% of the Nil.

### Statistical Methods

Variability in test interpretations was assessed by calculating the percentage of subjects with any discordance among the four ELISAs. Additionally, positive agreement, negative agreement, and agreement beyond chance (Cohen’s kappa statistic, *k*) were calculated for each pair of ELISAs. To assess variability in IFN-γ measurements (i.e., Nil, TB, and TB Response), distributions were compared using the Wilcoxon signed-rank test. Five additional indices of quantitative variability were examined for each pair of ELISAs, the last two of which were derived from the standard deviation of the differences (SD_diff_): (*1*) within-subject coefficient of variation (W-S CV%), (*2*) intraclass correlation coefficient (ICC), (*3*) mean difference (bias), (*4*) the smallest detectable difference (SDD), and (*5*) the within-subject standard deviation (W-S SD). SDD = 1.96*SD_diff_, and is the smallest change in a subsequent measurement that must occur to detect a change beyond the variability (e.g., noise) with 95% certainty [33], [34], W-S SD = ±(SD_diff_/√2) [35], and represents 68% of the variation expected around the true value [36]. Limits of agreement (LOA) = bias ± SDD and encompass the range around the bias that contains 95% of within-subject differences [37]. ICCs were calculated using the SAS macro ICC_SAS [38]. W-S CV% was calculated as described by Bland (root mean square approach) [39] for Nil and TB and estimated for TB Response using the formula √((W-S CV%_TB_)^2^+ (W-S CV%_Nil_)^2^) (root sum square method for estimating aggregate uncertainty). The W-S CV%s for the TB Response could not be directly determined due to inflation caused by zeros and negative mean values in the denominator (because some TB Response values were ≤0). A confidence level of 0.95 was used in all hypothesis tests. Stratified analyses for quantitative indices were performed on groups stratified by mean TB Response from all four ELISAs. SAS v9.2 (SAS, Cary, NC) and “Analyse-It” v2.22 for Excel (Analyse-It Software, Ltd., Leeds, UK) were used to perform the analyses.

## Results

### Subject Characteristics

**Figure 1:**
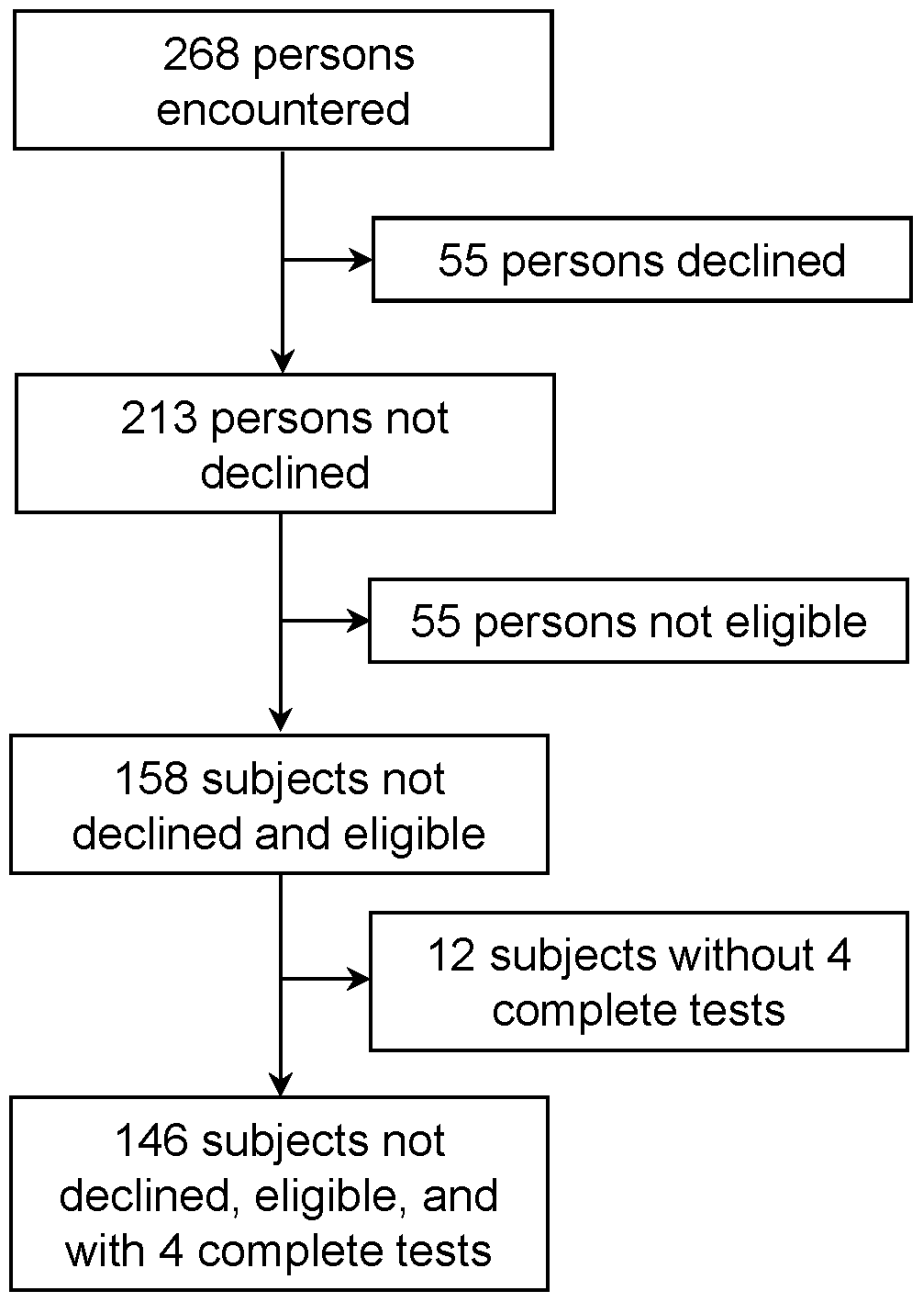
Study participation diagram.

Study participation is depicted in Figure 1. Of the 268 people asked to participate, 55 declined and 55 were not eligible. Of the 158 persons enrolled, 146 had four ELISAs completed (one automated and one manual ELISA for the first set of QFT-GIT tubes, and one automated and one manual ELISA for the second set of QFT-GIT tubes, referred to as A1, M1, A2, and M2, respectively). Characteristics of the study subjects are shown in Table 1.

**Table 1:** Subject characteristics.

Characteristic	Category	n (%)
Age, yr	20–29	16 (11.0%)
	30–39	32 (21.9%)
	40–49	41 (28.1%)
	50–59	37 (25.3%)
	≥60	20 (13.7%)
Gender	M	65 (44.5%)
	F	81 (55.5%)
Race/Ethnicity	White, non-Hispanic	70 (48.0%)
	Black, non-Hispanic	36 (24.7%)
	Asian/Pacific	18 (12.3%)
	Hispanic	13 (8.9%)
	Native American	1 (0.7%)
	Other	8 (5.5%)
Year of Last Positive TST	1950–1959	1 (0.7%)
	1960–1969	8 (5.5%)
	1970–1979	10 (6.9%)
	1980–1989	16(11.0%)
	1990–1999	60 (41.1%)
	2000–2009	51 (34.9%)
Received Therapy for TB	Yes	3 (2.1%)
	No	143 (98.0%)
Received Therapy for LTBI	Yes	106 (72.6%)
	No/Unknown	40 (27.4%)
Known Exposure to Active TB	Yes	55 (37.7%)
	No/Unknown	91 (62.3%)
Received BCG Vaccine	Yes	30 (20.5%)
	No/Unknown	116 (79.5%)
Region of Birth	United States andCanada	103 (70.5%)
	Asia	14 (9.6%)
	Central America/Caribbean	12 (8.2%)
	Africa	6 (4.1%)
	Europe/Russia	4 (2.7%)
	Pacific	3 (2.1%)
	Southeast Asia	2 (1.4%)
	Middle East	2 (1.4%)
Years Lived Outside USA	None	62 (42.5%)
	1–10	56 (38.4%)
	11–20	13 (8.9%)
	21–30	12 (8.2%)
	31–40	3 (2.1%)

### Qualitative Results

QFT-GIT interpretations are summarized by ELISA method and tube set in Table 2. Among the four tests, interpretations were concordantly positive for 24 (16%) subjects, concordantly negative for 106 (72.6%) subjects, and discordant for 16 (11%) subjects. Forty subjects (27.4%) had at least one positive interpretation. Two subjects (1.4%) had three positive interpretations, eight subjects (5.5%) had two positive interpretations, and five subjects (3.4%) had one positive interpretation. One subject had three indeterminate interpretations with low Mitogen Responses of 0.249 to 0.474 IU/mL and one negative interpretation with a Mitogen Response of 0.55 IU/mL. Nil, TB, and TB Response values for the 15 subjects with discordant results among the four tests (excluding the one subject with three indeterminate results) are shown in Table 3. Results are grouped as either single discordant (one discordant/three concordant) or double discordant (two opposing pairs of concordant results) and additionally categorized into eight groups according to the specific nature of the discordance. Twelve subjects (categories 1–6) were discordant between first and second tests. Two subjects had both automated tests positive and both manual tests negative (category 7), and one had both automated tests negative and both manual tests positive (category 8).

**Table 2:** QFT-GIT interpretations when ELISAs were performed with automated and manual methods.

Result	Automated 1	Automated 2	Manual 1	Manual 2
Positive	29 (19.9%)	30 (20.6%)	33 (22.6%)	31 (21.2%)
Negative	117 (80.1%)	115 (78.8%)	112 (76.7%)	114 (78.1%)
Indeterminate	0	1 (0.7%)	1 (0.7%)	1 (0.7%)

**Table 3:** Fifteen subjects discordant among any of the four tests.

		Automated 1				Automated 2				Manual 1				Manual 2			
Category*	ID	TB	Nil	TBResp.	Interp.	TB	Nil	TBResp.	Interp.	TB	Nil	TBResp.	Interp.	TB	Nil	TBResp.	Interp.
Single Discordant																	
1	35	0.363	0.153	0.210	Neg	0.439	0.147	0.292	Neg	0.490	0.125	0.365	Pos	0.289	0.112	0.177	Neg
1	113	0.228	0.310	−0.082	Neg	0.149	0.100	0.049	Neg	0.970	0.549	0.421	Pos	0.355	0.360	−0.005	Neg
1	127	0.519	0.234	0.285	Neg	0.527	0.279	0.248	Neg	1.497	0.359	1.138	Pos	0.581	0.447	0.134	Neg
1	133	0.140	0.097	0.043	Neg	0.092	0.090	0.002	Neg	2.120	0.413	1.707	Pos	0.762	0.469	0.293	Neg
2	104	0.076	0.082	−0.006	Neg	0.125	0.105	0.020	Neg	0.860	0.611	0.249	Neg	1.127	0.514	0.613	Pos
3	96	0.366	0.035	0.331	Neg	0.612	0.073	0.539	Pos	0.509	0.066	0.443	Pos	0.766	0.069	0.697	Pos
4	63	0.775	0.184	0.591	Pos	0.496	0.171	0.325	Neg	0.685	0.206	0.479	Pos	0.555	0.186	0.369	Pos
Double Discordant																	
5	32	0.363	0.166	0.197	Neg	0.559	0.171	0.388	Pos	0.361	0.208	0.153	Neg	0.725	0.262	0.463	Pos
5	129	0.338	0.046	0.292	Neg	0.675	0.044	0.631	Pos	0.336	0.143	0.193	Neg	1.055	0.117	0.938	Pos
5	136	0.440	0.116	0.324	Neg	0.633	0.075	0.558	Pos	0.903	0.867	0.036	Neg	3.943	0.804	3.139	Pos
6	100	0.589	0.186	0.403	Pos	0.388	0.138	0.250	Neg	0.678	0.164	0.514	Pos	0.452	0.133	0.319	Neg
6	135	1.934	0.107	1.827	Pos	0.068	0.075	−0.007	Neg	2.322	0.288	2.034	Pos	0.317	0.502	−0.185	Neg
7	78	0.651	0.080	0.571	Pos	0.542	0.078	0.464	Pos	0.337	0.078	0.259	Neg	0.267	0.085	0.182	Neg
7	101	1.163	0.306	0.857	Pos	1.051	0.145	0.906	Pos	1.466	1.637	−0.171	Neg	1.383	1.185	0.198	Neg
8	102	0.131	0.053	0.078	Neg	0.145	0.079	0.066	Neg	0.599	0.195	0.404	Pos	0.847	0.381	0.466	Pos

* (1) 1st manual positive/others negative, (2) 2nd manual positive/others negative, (3) 1st automated negative/others positive, (4) 2nd automated negative/others positive, (5) 1st test negative/2nd test positive, (6) 1st test positive/2nd test negative, (7) automated positive/manual negative, (8) automated negative/manual positive.

**Figure 2:**
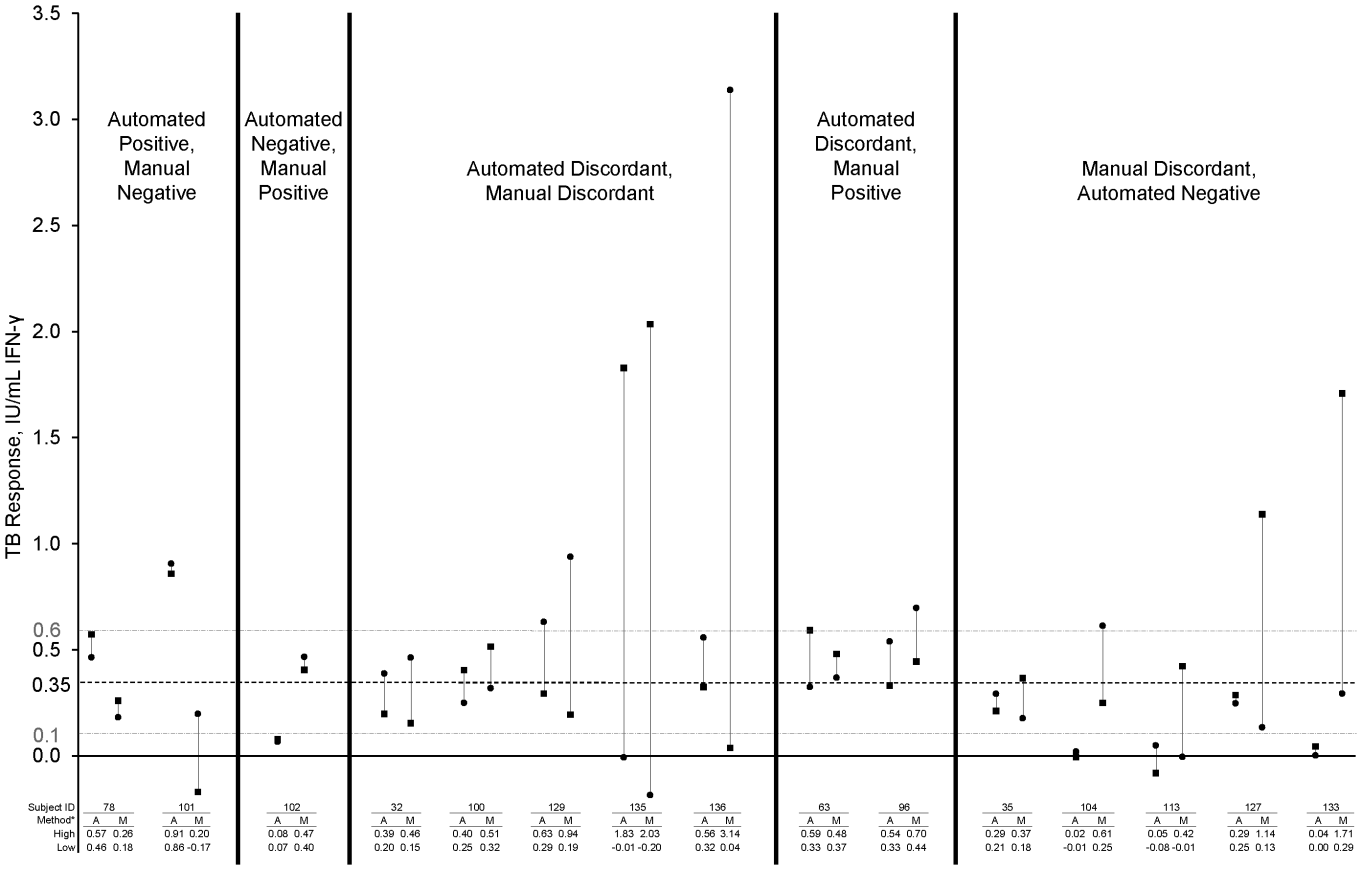
Comparison of TB Responses for subjects with discordant test interpretations. *A = automated, M = manual; squares = first test, circles = second test; 0.35 IU/mL cutoff shown by black dashed line; 0.1 to 0.6 IU/mL borderline zone (0.35±0.25 IU/mL) shown by grey dot-dashed line.

Indices of interpretation variability between pairs of ELISAs are shown in Table 4. Seven (4.8%) subjects had discordant results with automated ELISAs compared to 10 (6.9%) subjects with manual ELISAs (p = 0.17). Results from the 15 subjects with discordant results are depicted in Figure 2. Five of the 7 subjects discordant with the two automated tests (71%) had both TB Responses within ±0.25 IU/mL of the QFT-GIT cutoff (0.1 to 0.6 IU/mL, gray dot-dashed lines) vs. 3 of 10 (30%) subjects discordant with the two manual tests.

**Table 4:** Variability in QFT-GIT interpretations between ELISAs.

					% Agreement			% Discordant	
Results Compared (Group 1 vs. Group 2)	Both Positive	Both Negative	Positive*/Negative	Negative*/Positive	Overall	Positive	Negative	Overall	Kappa
A1 vs. A2	26	112	3	4	95.2	78.8	94.1	4.8	0.85
M1 vs. M2	27	108	6	4	93.1	73.0	91.5	6.9	0.80
A1 vs. M1	27	110	2	6	94.5	77.1	93.2	5.5	0.84
A1 vs. M2	25	110	4	6	93.1	71.4	91.7	6.9	0.73
A2 vs. M1	25	107	5	8	91.0	65.8	89.2	9.0	0.74
A2 vs. M2	28	112	2	3	96.6	84.8	95.7	3.4	0.90

* Group 1/Group 2; subject with indeterminate results not included.

### Quantitative Results

**Figure 3:**
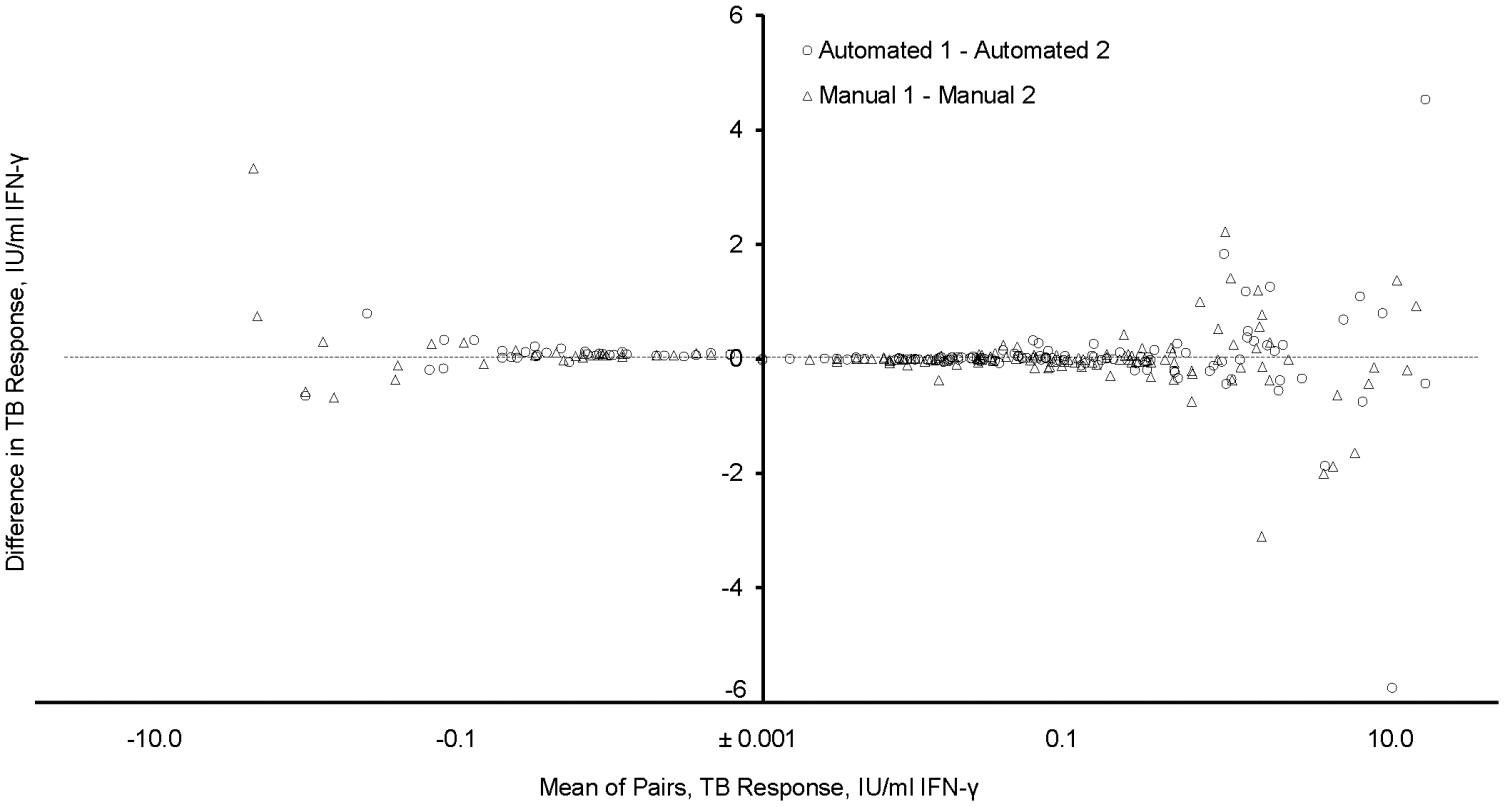
Bland-Altman plot of TB Responses. X- axis (mean of paired TB Responses) shown on log scale. Points between 0 to +0.001 and 0 to −0.001 not shown.

Means, medians, and ranges for Nil, TB, and TB Response are shown in Table 5. There were no significant distributional differences between the two automated tests or between the two manual tests, but TB and NIL values in manual tests were significantly greater than in automated tests (p<0.03). There were no significant differences in TB Response between manual and automated tests. ICCs and W-S CV%s are shown in Table S1. Examination of difference (Bland-Altman) plots for TB Response, shown in Figure 3, shows an increase in variation as the mean of the paired measurements increased.

**Table 5:** IFN-γ means, medians, and ranges for the four tests (IU/mL).

	TB			Nil			TB Response		
Test	Mean	Median	Range	Mean	Median	Range	Mean	Median	Range
A1	0.89	0.12	0.03 to 20.17	0.12	0.08	0.03 to 1.99	0.77	0.05	−1.19 to 20.04
A2	0.87	0.12	0.04 to 18.07	0.11	0.07	0.03 to 1.43	0.76	0.02	−0.69 to 17.99
M1	0.91	0.18	0.04 to 17.88	0.21	0.12	0.03 to 1.71	0.70	0.03	−0.83 to 17.78
M2	0.92	0.19	0.05 to 16.11	0.22	0.11	0.04 to 1.71	0.70	0.03	−1.36 to 15.99

**Figure 4:**
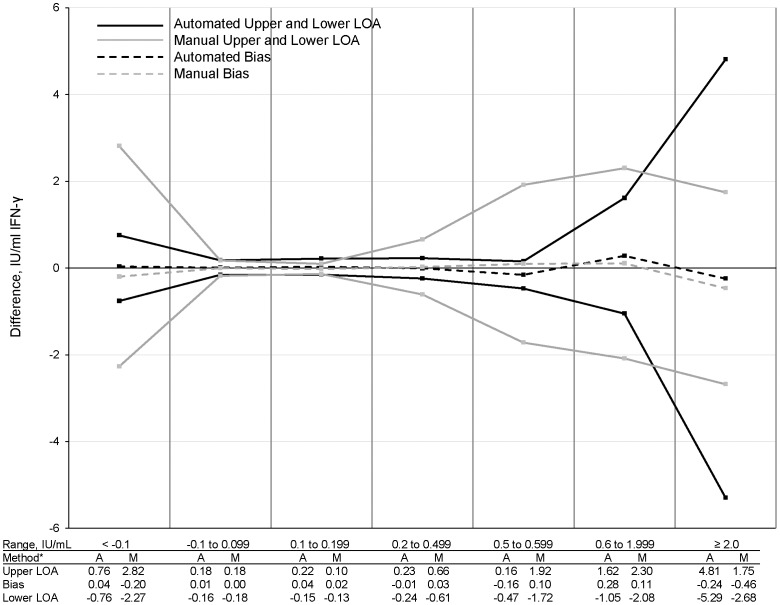
95% Limits of agreement and bias in TB Responses. *A = automated, M = manual.

Analyses were performed examining variation within seven strata of mean TB Response, based on the mean of the four tests. Bias and 95% LOA are shown in Figure 4. The relatively large variability seen for the first stratum (<0.1 IU/mL) is due to grouping subjects with negative means, many of whom had large differences (which also may be seen in Figure 3). The fourth stratum (0.2 IU/mL to 0.499 IU/mL) shows variability in a range surrounding the QFT-GIT cutoff (0.35±0.15 IU/mL). In this category, bias and LOA for manual tests were greater than for automated tests. As shown in Table S2, significantly higher W-S SDs were observed within this range for manual tests than for automated tests, as demonstrated by non-overlapping 95% confidence intervals (95% CI). SDDs for this range were also significantly higher for the manual tests than for the automated tests. When this range was expanded to 0.1 IU/mL to 0.6 IU/mL (0.35±0.25 IU/mL), W-S SDs remained significantly higher for manual tests (0.27, 95% CI: 0.22–0.37) than for automated tests (0.09, 95% CI: 0.07–0.12). SDDs were also significantly higher for manual tests (0.75, 95% CI: 0.61–1.03) than for automated tests (0.25, 95% CI: 0.19–0.33) for this broader range.

## Discussion

This study assessed the precision of the QFT-GIT using both automated and manual ELISA methods. We determined repeatability of QFT-GIT when performed manually on two blood samples collected at the same time and when performed with the aid of an automated ELISA workstation on two blood samples collected at the same time. We observed discordance of 4.8% between two automated tests and 6.9% between two manual tests. Additionally, we evaluated reproducibility of QFT-GIT when one test was performed manually and one test was performed with the aid of an automated ELISA workstation on blood samples collected at the same time. We observed discordance of 3.4% to 9.0% for automated versus manual paired combinations. Eleven percent of subjects (including the one subject with one negative result and three indeterminate results) had at least one discordant result among the four tests. Quantitative indices of variability showed that variation in TB Response near the cutoff separating positive and negative test interpretations was significantly greater with the manual method than with the automated method.

Our discordance rates of 4.8% for two repeated automated QFT-GITs and 6.9% for two repeated manual QFT-GITs are slightly higher than those from two similar studies in which ELISAs were repeated on blood collected at the same time [2], [10]. Discordant rates of 3.6% were reported in both studies; however, the ELISA methods used for these studies were not described. QFT-GIT is a complex assay, but investigators rarely specify details for performing the ELISAs.

Our estimates of QFT-GIT reproducibility when performed in the same lab using automated or manual methods ranged from 3.4% to 9.0%. Prior estimates of QFT-GIT reproducibility when ELISAs were performed in different labs using automated methods ranged from 3.3% to 6.6% [21]. Our finding of greater variability when the QFT-GIT ELISA is performed manually than when performed with the aid an automated ELISA workstation is not surprising, given the complexity of the assay. In a prior study, we reported that a reduction in the number of steps required for QFT-GIT compared to QFT-G was associated with a significant reduction in the number of unusual measurements [19].

We and others have previously suggested the need for a zone of uncertainty surrounding the 0.35-IU/mL cutoff currently used to separate positive and negative QFT-GIT results [2]–[4], [6], [13], [21], [40]. Clinicians may need to repeat testing when initial results are within a borderline zone to increase diagnostic certainty. However, there is no consensus on the size of the zone, and different sizes have been suggested or applied. Our finding of greater variability when the QFT-GIT ELISA is performed manually than when aided by an automated workstation suggests that a broader borderline zone would be needed when using manual methods. Use of a broader borderline zone may, in turn, necessitate more repeat testing. Greater precision may justify the cost of an automated ELISA workstation.

Our study has several limitations. First, the small sample size for some strata resulted in large confidence intervals for estimates of variability. Despite the small sample size, differences in variability between automated and manual TB Response in the stratum surrounding the cutoff were significant. Second, we only studied TB Response in persons who reported a prior positive TST. While other populations may have different proportions of negative, positive, and borderline TB Response values, this limitation would not be expected to alter variability within strata of TB Response values.

In conclusion, automation of QFT-GIT ELISA may reduce variability near the cutoff separating positive or negative interpretations. Methodological differences should be considered when interpreting and using IGRAs.

## Supporting Information

Table S1 ICC and W-S CV%, total population. *95% confidence interval.
(DOC)

Table S2 W-S SD and SDD for TB Response, total population and stratified. *95% confidence interval.
(DOC)
