# Supplementary material for: Variability of the QuantiFERON®-TB Gold In-Tube Test Using Automated and Manual Methods
Source: PLoS One. 2014 Jan 23;9(1):e86721. doi: 10.1371/journal.pone.0086721 (PMC3900587; doi:10.1371/journal.pone.0086721)
Supplement: Table S1 — ICC and W-S CV%, total population. *95% confidence interval. (DOC) [file pone.0086721.s001.doc]

**TABLE S1. ICC and W-S CV%, total population**

| Measure | Comparison | ICC (95% CI*) | W-S CV% (95% CI) |
| --- | --- | --- | --- |
|  |  |  |  |
| Nil | A1 vs. A2 | 0.80 (0.73, 0.85) | 27.46 (22.41, 31.72) |
|  | M1 vs. M2 | 0.65 (0.55, 0.73) | 26.13 (20.82, 30.54) |
|  |  |  |  |
| TB | A1 vs. A2 | 0.96 (0.95, 0.97) | 26.51 (20.54, 31.36) |
|  | M1 vs. M2 | 0.98 (0.97, 0.99) | 28.55 (23.43, 32.89) |
|  |  |  |  |
| TB Response | A1 vs. A2 | 0.96 (0.95, 0.97) | 38.16 (30.40, 44.60) |
|  | M1 vs. M2 | 0.97 (0.96, 0.98) | 38.71 (31.34, 44.88) |

*95% confidence interval
