# Supplementary material for: Variability of the QuantiFERON®-TB Gold In-Tube Test Using Automated and Manual Methods
Source: PLoS One. 2014 Jan 23;9(1):e86721. doi: 10.1371/journal.pone.0086721 (PMC3900587; doi:10.1371/journal.pone.0086721)
Supplement: Table S2 — W-S SD and SDD for TB Response, total population and stratified. *95% confidence interval. (DOC) [file pone.0086721.s002.doc]

**TABLE S2.** W-S SD and SDD for TB Response, total population and stratified.

| Comparison | Strata | n | W-S SD (95% CI*) | SDD (95% CI) |
| --- | --- | --- | --- | --- |
|  |  |  |  |  |
| A1 vs. A2 | total | 146 | 0.49 (0.44, 0.56) | 1.36 (1.22, 1.55) |
|  | < -0.1 | 8 | 0.04 (0.02, 0.07) | 0.11 (0.06, 0.19) |
|  | -0.1 to 0.099 | 84 | 0.06 (0.05, 0.07) | 0.17 (0.14, 0.19) |
|  | 0.1 to 0.199 | 10 | 0.07 (0.05, 0.12) | 0.19 (0.14, 0.33) |
|  | 0.2 to 0.499 | 15 | 0.08 (0.06, 0.13) | 0.22 (0.17, 0.36) |
|  | 0.5 to 0.599 | 4 | 0.11 (0.06, 0.42) | 0.30 (0.17, 1.16) |
|  | 0.6 to 1.999 | 15 | 0.48 (0.35, 0.76) | 1.33 (0.97, 2.11) |
|  | ≥ 2.0 | 10 | 1.82 (1.25, 3.33) | 5.04 (3.46, 9.23) |
|  |  |  |  |  |
| M1 vs. M2 | total | 146 | 0.41 (0.37, 0.46) | 1.14 (1.03, 1.28) |
|  | < -0.1 | 8 | 0.11 (0.07, 0.22) | 0.30 (0.19, 0.61) |
|  | -0.1 to 0.099 | 84 | 0.06 (0.06, 0.08) | 0.17 (0.17, 0.22) |
|  | 0.1 to 0.199 | 10 | 0.04 (0.03, 0.08) | 0.11 (0.08, 0.22) |
|  | 0.2 to 0.499 | 15 | 0.23 (0.17, 0.36) | 0.64 (0.47, 1.00) |
|  | 0.5 to 0.599 | 4 | 0.66 (0.37, 2.44) | 1.83 (1.03, 6.76) |
|  | 0.6 to 1.999 | 15 | 0.79 (0.58, 1.25) | 2.19 (1.61, 3.46) |
|  | ≥ 2.0 | 10 | 0.80 (0.55, 1.46) | 2.22 (1.52, 4.05) |

*95% confidence interval
